# Growth and Functionalization of Particle-Based Mesoporous Silica Films and Their Usage in Catalysis

**DOI:** 10.3390/nano9040562

**Published:** 2019-04-06

**Authors:** Pei-Hsuan Wu, Peter Mäkie, Magnus Odén, Emma M. Björk

**Affiliations:** 1 Nanostructured Materials, Department of Physics, Chemistry and Biology (IFM), Linköping University, 581 83 Linköping, Sweden; peiwu389@student.liu.se (P.-H.W.); p_makie@hotmail.com (P.M.); magnus.oden@liu.se (M.O.); 2 Institute of Inorganic Chemistry II, University of Ulm, Albert-Einstein-Allee 11, 890 81 Ulm, Germany

**Keywords:** mesoporous silica, mesoporous films, direct growth, esterification, material formation

## Abstract

We report the formation of mesoporous films consisting of SBA-15 particles grown directly onto substrates and their usage as catalysts in esterification of acetic acid and ethanol. The film thickness was altered between 80 nm and 750 nm by adding NH_4_F to the synthesis solution. The salt also affects the formation rate of the particles, and substrates must be added during the formation of the siliceous network in the solution. Various substrate functionalizations were tested and hydrophobic substrates are required for a successful film growth. We show that large surfaces (> 75 cm^2^), as well as 3D substrates, can be homogenously coated. Further, the films were functionalized, either with acetic acid through co-condensation, or by coating the films with a thin carbon layer through exposure to furfuryl alcohol fumes followed by carbonization and sulfonation with H_2_SO_4_. The carbon-coated film was shown to be an efficient catalyst in the esterification reaction with acetic acid and ethanol. Due to the short, accessible mesopores, chemical variability, and possibility to homogenously cover large, rough surfaces. the films have a large potential for usage in various applications such as catalysis, sensing, and drug delivery.

## 1. Introduction

Mesoporous silica films are of large interest in applications such as sensing, catalysis, and drug delivery [1,2,3,4,5,6]. Their large surface area, tunable pore characteristics, and versatile surface functionality are attractive features. For some applications, ordered cylindrical pores are preferable over spherical [7] or wormlike [8] pores. SBA-15 is a type of mesoporous silica with hexagonally ordered, cylindrical pores [9]. By alterations in the synthesis conditions, e.g. addition of swelling agents or altered reaction temperature, the pores can be increased from the regular ~8 nm to > 18 nm [10,11], and the particle morphology changed from fibers to rods or platelets [11,12]. This makes the material attractive in, e.g., catalysis [13,14], enzyme immobilization [15], and sensing [16]. However, the synthesis of mesoporous films using SBA-15 structures most often results in long pores that are aligned parallel to the substrate [17], making them inaccessible.

The most common method for synthesizing SBA-15 films is evaporation-induced self-assembly (EISA) [18], where an ethanol-containing solution is deposited onto a substrate using spin- or dip-coating. The method can be used on large substrates with various compositions, but is limited, as the dipping angle is crucial for the film thickness [19], resulting in non-homogenous coatings on non-flat substrates, and pores larger than 10 nm are rare [20]. Recently, methods have been developed for depositing thin layers of pre-synthesized mesoporous silica particles on substrates, e.g., by spinning them onto a substrate [21], or using Langmuir-Blodgett deposition [22], making also cylindrical pores easily accessible. However, these films consist of separated particles located on the substrate, which results in poor mechanical integrity. In addition, the method requires flat substrates, which makes it less attractive for many applications. It is possible to bind the particles to a substrate through covalent linking [5], but this is a time-consuming procedure, with functionalization of both particles and substrates. A different direct growth (DiG) method, to form a monolayer of SBA-15 particles adhered to a substrate using a simple one-pot synthesis method, was recently reported [23]. This method yields densely packed, separate particles with short, accessible pores for gases and liquids, despite having the pores running parallel to the substrate surface.

In the present study, we show that mesoporous films with controlled thickness can be synthesized by growing monodispersed SBA-15 particles onto substrates. The effect of substrate functionalization, as well as the formation of the films, are investigated, and we show that it is possible to form mechanically stable films on large and rough surfaces. Functionalization of the films using co-condensation and the possibility to coat the films with a carbon layer through exposure of furfuryl alcohol vapor, forming CMK-5 [24], are investigated to study the variability of the films. To explore the accessibility of the pores, the film is tested as a catalyst for esterification of acetic acid and ethanol. The DiG-technique can be suitable for synthesizing mesoporous coatings for, e.g., catalysts, sensors, or medical implants.

## 2. Materials and Methods

### 2.1. Reagents

Hydrochloric acid (≥37%, puriss. p.a., Fluka, ACS Reagent, fuming), triblock copolymer EO20PO70EO20 (P123) (Mn ~5800, Aldrich, ammonium fluoride (≥ 98.0%, puriss. p.a., ACS reagent, Fluka), tetraethyl orthosilicate (TEOS)(reagent grade, 98%, Aldrich), heptane (99%, ReagentPlus®, Sigma-Aldrich), (3-Mercaptopropyl)trimethoxysilane (MPTMS) (Aldrich), hydrogen peroxide (≥35% at RT, purum p.a., Sigma-Aldrich), octadecyltrichlorosilane (OTS) (≥ 90%, Aldrich), chlorotrimethylsilane (TMCS) (≥ 99%, Aldrich), (3-Aminopropyl)trimethoxysilane (APTMS) (97%, Aldrich), toluene (≥ 99.5%, Sigma-Aldrich), nitric acid (≥ 64–66%, Sigma-Aldrich), sulphuric acid (95.0–98.0%, ACS Reagent, Sigma-Aldrich), glycerol (≥ 99.0%, Sigma-Aldrich), acetone (≥99.9%, Sigma-Aldrich), 1,6-diisocynanatohexane (98%, Aldrich), ethanol (95%, Kemetyl), ethanol (99.5 %, Solveco), furfuryl alcohol (98%, Aldrich), methanol (99.8%, Sigma-Aldrich), and benzene (purity ≥ 99.7%, Sigma-Aldrich)) were purchased from Sigma-Aldrich, Stockholm, Sweden, and used as received.

### 2.2. Syntheses

#### 2.2.1. Functionalization of Substrates

OTS-functionalized Si wafers were used as substrates for the DiG films [23]. Prior to functionalization, organic contaminants on the substrates were removed from the surface with standard Radio Corporation of America (RCA) cleaning fluid (H_2_O:H_2_O_2_:NH_3_ in a volume ratio of 5:1:1 at 85 ℃ for 10 min), washed with water, and then treated with nitric acid at room temperature for 5 min. The substrates were then washed with large amounts of water and dried with compressed N_2_ gas. The OTS was grafted onto the substrates by immersing them in a heptane solution with 1 mM OTS at 18 °C for 15 min. After the OTS treatment, the substrates were rinsed with heptane, dried at 200 °C for 2 h, and stored in heptane until usage.

Details of the additional functionalization methods used are presented in the Supplementary Materials.

#### 2.2.2. Film Synthesis

The films were grown following a modified protocol from Björk et al. [23]. In a typical synthesis, 2.4 g of P123 and a specific amount (0–28 mg) of NH_4_F were dissolved in 80 ml of 1.84 M HCl solution at 20 °C. The mixture was stirred in a round bottom flask until the reagents were dissolved. 5.5 ml of TEOS and 1 ml heptane were premixed and added to the micellar solution under stirring for 4 min, followed by static conditions overnight. During this static time, substrates were immersed in the solution at specific time intervals, depending on the amount of NH_4_F used. The substrates were transferred to a sealed polytetrafluorethylene (PTFE) flask, while still being submersed in the solution, and placed in an oven at 100 °C for 24 h for hydrothermal treatment. The products were collected by filtration, rinsed with deionized water, and dried at ambient temperature overnight. For template removal, both the films and powder were calcined in air at 550 °C for 5 h with a 2 °C/min ramp rate. The materials are labelled SBA-15_*X*, where *X* is the NH_4_F/P123 molar ratio.

#### 2.2.3. Direct Sulfonation (SBA-15-DS)

Films with sulfonic acid groups were synthesized by adding MPTMS and H_2_O_2_ during the particle formation, following the protocol by Margolese et al. [25]. A double batch of SBA-15_0.0 was synthesized, and when the stirring was turned off, the solution was divided into six beakers. In five beakers, 0.17 mL of MPTMS and 0.27 mL of H_2_O_2_ were added to the synthesis solution at different times (1–20 h) after the TEOS addition. These materials are labelled SBA-15_0.0_*Y*DS, where *Y* corresponds to the hours between the addition of TEOS and MPTMS + H_2_O_2_. For these materials, the P123 was removed by ethanol extraction, as described in the Supplementary Materials.

#### 2.2.4. Carbon Infiltration and Sulfonation (SBA-15-CMK-5)

To get an even distribution of carbon species in the films, the carbon infiltration was performed by exposing the silica films to furfuryl alcohol vapor. Calcined SBA-15_0.0 was exposed to alcohol vapor from furfuryl alcohol (5 ml) in a closed atmosphere at 40 °C overnight. The composites were then kept in a furnace at 100 °C overnight to ensure an even distribution of furfuryl alcohol into the mesopores, and to polymerize the alcohol. The material was then transferred to a nitrogen purged tube furnace at 800 °C for 1 h. Pyrolysis of the polymer during the heat treatment resulted in a thin carbon layer on the mesoporous silica walls.

The carbon infiltrated SBA-15 was sulfonated according to previous reports [13,26]. Briefly, 0.5 g of SBA-15-CMK-5 was mixed with 25 ml of H_2_SO_4_ and heated to 80 °C under reflux overnight. After the reaction, the solution was cooled down to room temperature, rinsed with large amounts of deionized water, and collected by filtration. The SBA-15-CMK-5-SO3H was finally dried at 80 °C overnight.

### 2.3. Characterization

The material morphology was observed by scanning electron microscopy (SEM), using a Leo 1550 Gemini Scanning Electron Microscope (Zeiss) operated at 3 kV and a working distance of 3–5 mm. The pore characteristics were determined for all powders using N_2_ sorption with an ASAP2020 (Micromeritics) at −196 °C. The specific surface area was determined with the BET method at *P*/*P*_0_ = 0.8–0.18, and the total pore volume was calculated at *P*/*P*_0_ = 0.98. The pore size was calculated using the KJS method at the adsorption isotherms. Small angle x-ray diffraction (SAXRD) was used to identify the pore order. Diffractograms were recorded with an Empyrean diffractometer from, in transmission mode using Cu K_α_ radiation (Malvern Panalytical). The pore structure was further visualized by transmission electron microscopy (TEM) performed with a Tecnai G2 TF 20 UT microscope operated at 200 kV (FEI). TEM samples were prepared by dispersing the product in acetone and depositing it on hollow carbon grids.

The contact angle of the functionalized substrates was determined by contact angle measurements using a CAM 200 Optical Contact Angle Meter (KSV Instruments). The measurements were performed using a 2 µL droplet of distilled water, which was placed in the middle of the substrate. Three independent measurements were conducted for each substrate.

Determination of sulfonic acid groups was performed using acid-base titration. For the silica-based samples, 0.10 g of the material was mixed with deionized water, followed by direct acid-base titration with a 0.005 M NaOH solution. For the SBA-15-CMK-5-SO3H, the catalyst was mixed with 30 ml 0.1 M Na_2_SO_4_ to react with the sulfonic groups, forming bisulfate, 4 h prior to the acid-base titration. The bisulfate reacted with the NaOH, giving rise to the total amount of sulfonic acid groups. Other acid groups (carboxylic, phenolic, lactonic) are less acidic than sulphate, and do not create bisulfate upon exposure to sulphate ions [27]. The number of acidic groups was calculated as

*n*_ac_ = *V*_NaOH_ × [NaOH] (1)

### 2.4. Esterification Reaction

The catalytic activity of an SBA-15-CMK-5-SO3H DiG film is shown in an esterification reaction with acetic acid and ethanol. In the reaction, 25 mL of ethanol and 10 mL of acetic acid was mixed and heated to 80 °C under stirring in a closed beaker. A 4-inch silicon wafer coated with SBA-15_CMK-5_SO3H cut in pieces was added to the mixture. 1 mL aliquots were removed from the solution at specific time points and mixed with deionized water to terminate the reaction. The conversion of acetic acid was determined by titration with a 1 M NaOH solution, and the equivalence point was found using a pH electrode. The conversion was calculated using

*X* (%) = (*mol*HAcinitial − *mol*HAcend)/*mol*HAcinitial × 100 (2)

As a reference, the reaction was also performed without the presence of a catalyst.

## 3. Results

### 3.1. Film Growth

**Figure 1:**
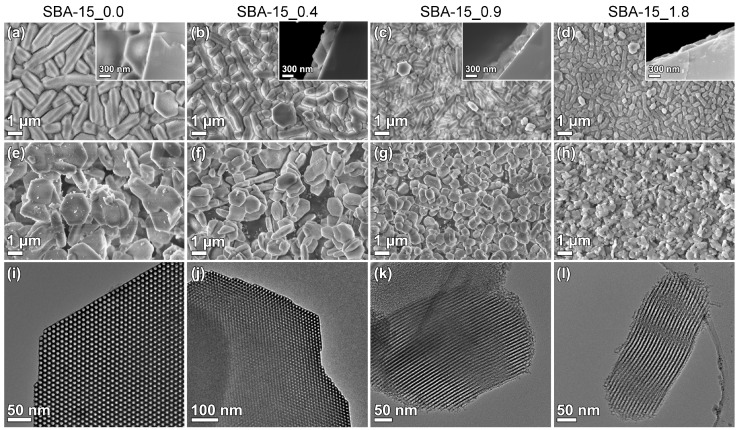
SEM micrographs of films (first row) and particles (second row), and TEM micrographs of particles (third row) synthesized with a NH_4_F to P123 molar ratio of (**a**,**e**,**i**) 0.0, (**b**,**f**,**j**) 0.4, (**c**,**g**,**k**) 0.9, and (**d**,**h**,**l**) 1.8.

Films were synthesized with various NH_4_F concentrations in the solution. The particle sizes, both on the substrates and in the solution, as well as film thickness, were affected by the salt concentration, as seen in Figure 1. It was clear that densely packed films could be grown independent of the NH_4_F concentration. The particle size, both on the substrate and in powder form, was affected by the salt concentration. The particles became narrower, from platelets (Figure 1e,f) to rods (Figure 1g,h), with an increasing NH_4_F to P123 molar ratio. The particle narrowing was consistent with the results from Björk et al. [12], and was a result of the decreased solubility of the polyethylene oxide (PEO) chains of P123 when the NH_4_F concentration increases. The TEM micrographs show cylindrical pores, ordered in a hexagonal structure, for all NH_4_F concentration. The pore orientation supported that side-by-side attachment of the micelles causes the particle broadening.

**Figure 2:**
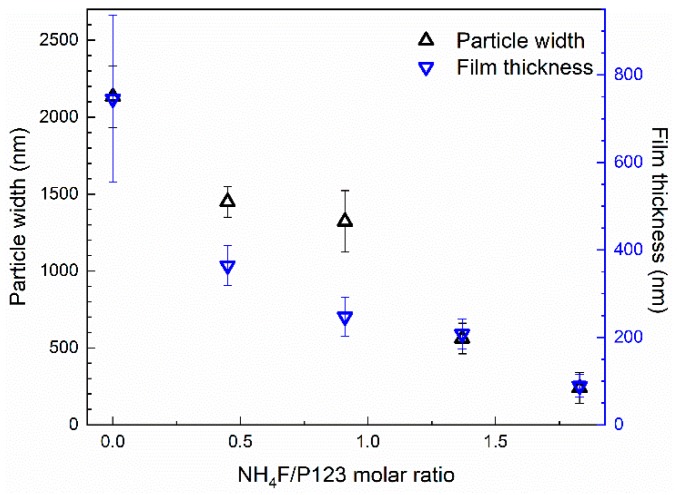
The relation between film thickness and particle width for different direct growth (DiG) films synthesized with various NH_4_F to P123 molar ratios.

The relation between the width of the film particles and the film thickness is presented in Figure 2. It is apparent that the film thickness and particle width follow the same trend, even though the film thickness is a factor of 2–3 times smaller than the particle width. Nitrogen sorption isotherms, SAXRD diffractograms, and the corresponding physicochemical properties of the SBA-15 powders from the film syntheses are available in the Supplementary Materials (Figure S1 and Table S1). These results show that the pores were cylindrical and ~10 nm in diameter. The x-ray diffractograms show three well-resolved peaks for all materials, confirming the hexagonal ordering of the pores.

**Figure 3:**
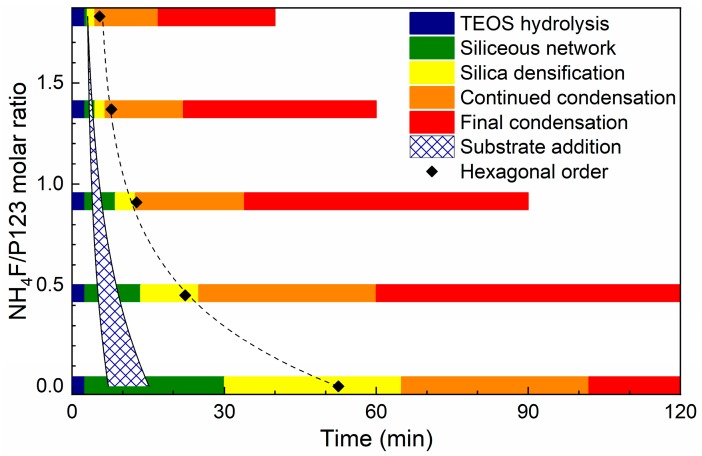
The window for substrate addition for a dense film growth and its correlation for material formation stages (Adapted from [28]).

To study the mechanism for a successful film growth, substrates were added at different times, depending on the formation rate. It is well known that the formation rate of SBA-15 is affected by addition of NH_4_F, where higher concentrations of salt give a faster formation rate [12,28]. The substrate addition times yielding the desired film morphology and its correlation to the formation stages of the material are presented in Figure 3. The films were evaluated by SEM, and successful film growth is here defined as a homogenous layer of densely packed particles on the substrate, see the first row in Figure 1.

### 3.2. Surfactant Removal

**Figure 4:**
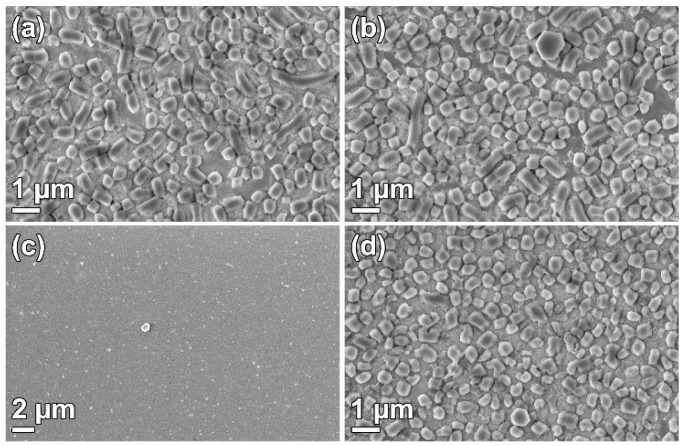
SEM micrographs of DiG_0.4 films where the surfactant was removed by (**a**) calcination, (**b**) ethanol extraction, (**c**) H_2_O_2_ oxidation, and (**d**) methanol sonication.

The choice of micelle removal technique can be used to tailor the material characteristics, e.g., pore size, silanol group concentration, or survival of co-condensed functional groups. Figure 4. shows SBA-15_0.4 films where the surfactant was removed with various methods: calcination, ethanol extraction [9], H_2_O_2_ oxidation [11], and methanol sonication [29]. The removal techniques are presented in the Supplementary Materials. It is clear from Figure 4a,b,d that surfactant removal by calcination, ethanol extraction, and methanol sonication did not affect the film morphology. However, H_2_O_2_ oxidation (Figure 4c) removed the particles from the substrate, resulting in a nearly naked substrate surface.

### 3.3. Substrate Effects

**Figure 5:**
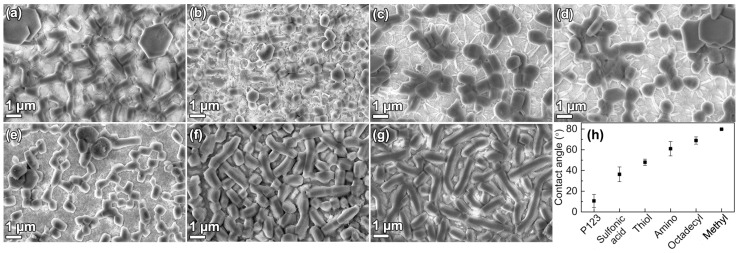
SEM micrographs of DiG_0.0 films grown onto substrates functionalized with (**a**) silanol groups (**b**) P123, (**c**) sulfonic acid, (**d**) thiol groups, (**e**) amino groups, (**f**) octadecyl groups, (**g**) methyl groups, and (**h**) the contact angle for the corresponding substrates.

Several types of substrate functionalizations were used here to study the requirements for film growth. The methods for functionalization of silicon wafers with P123, sulfonic acid, thiol groups, amino groups, octadecyl groups, and methyl groups are presented in the Supplementary Materials. The contact angles for the films and SEM micrographs of the grown films are presented in Figure 5. No contact angle value is presented for the clean wafer with silanol groups, since it was so hydrophilic that no angle could be determined. As can be seen, dense DiG films (SBA-15_0.0) could only be grown on substrates functionalized with octadecyl or methyl groups (Figure 5f,g), which are the most hydrophobic substrates. The silanol and P123 functionalized substrates also held a number of small SBA-15 particles (Figure 5a,b), while the substrate with sulfonic acid, thiol, and amino groups mainly consisted of a tissue phase and some particles (Figure 5c–e).

**Figure 6:**
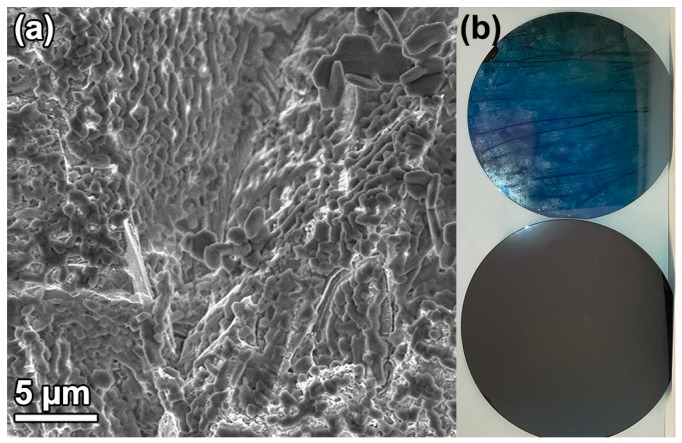
(**a**) SEM micrograph of a SBA-15_0.0 DiG film grown on a blasted substrate, and (**b**) a photograph of (top) a SBA-15_0.0coated and (bottom) clean 4 inch silicon wafer.

Figure 6 shows that it was possible to grow films with the DiG method on rough and large area substrates. An SBA-15_0.0 film was grown on a silicon wafer that was blasted with alumina sand, creating a rough surface, prior to the OTS functionalization. Figure 6a shows a dense film coverage of the rough substrate surface, where particles are grown on all surfaces, independent of incline. Also, a full 4-inch silicon wafer was coated with SBA-15_0.0, resulting in a homogenous film across the substrate (Figure 6b). The substrate addition time for both syntheses was 16 min after TEOS addition.

### 3.4. Functionalization

**Figure 7:**
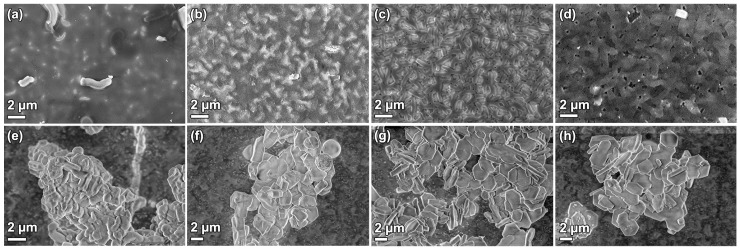
SEM micrographs of SBA-15_0.0 films (first row) and particles (second row) functionalized by co-condensation (**a**,**e**) 1 h, (**b**,**f**) 2 h, (**c**,**g**) 4 h, and (**d**,**h**) 20 h after the addition of TEOS.

SBA-15_0.0 films were functionalized with sulfonic acid using MPTMS and H_2_O_2_ during a co-condensation process during the material formation. The SBA-15_0.0 synthesis was chosen since it has the lowest formation rate, and a mixture of MPTMS and H_2_O_2_ was added to the synthesis mixture after 1, 2, 4, and 20 h into the reaction. The morphology of the functionalized particles and films are shown in Figure 7 and it can be observed that the addition time affected the material characteristics. When the MPTMS/H_2_O_2_ was added 1 h into the synthesis, the particles were small and aggregated resulting in inhomogeneous coverage of the substrate surface. The film consists of a tissue phase with sparsely attached particles (Figure 7b). When the reagents were added after 2 h, the platelet morphology had started to form (Figure 7c), but the particles were narrower compared to the original SBA-15_0.0, and in addition, small spherical features coexisted. The corresponding films consisted of particles, but it was apparent that these were smaller and not as developed as the unfunctionalized films in Figure 1a. When the MPTMS/H_2_O_2_ was added after 4 h, both the particle and film morphologies (Figure 7e,f) resembled the unfunctionalized materials. Finally, when the functionality was added after 20 h, i.e., directly prior to the hydrothermal treatment, the particle morphology was unaffected, but the appearance of the films was fuzzy, as if the particles had been covered with an additional layer (Figure 7g,h).

The acidity and physicochemical properties of the materials are presented in Table 1. Nitrogen sorption isotherms and small angle x-ray diffractograms are provided in the Supplementary Materials (Figure S2). The small angle x-ray diffractograms show three peaks, confirming a hexagonal order of the pores, also for the sulfonated materials. The less intense 110 and 200 peaks of SBA-15_1DS indicate a lower degree of order compared to when functionalization was performed later in the synthesis. The highest numbers of acidic groups were found in the materials functionalized after 1 and 4 h after the TEOS addition with 0.024 mmol/g and 0.020 mmol/g, respectively, compared to 0.003 mmol/g without functionalization. The addition of the reagents after 2 h and prior to the hydrothermal treatment had a negligible effect on the acidity of the materials compared to unfunctionalized SBA-15_0.0. It should be noted that the acidity was only measured on the powder materials, due to the small material amount on the substrates.

**Figure 8:**
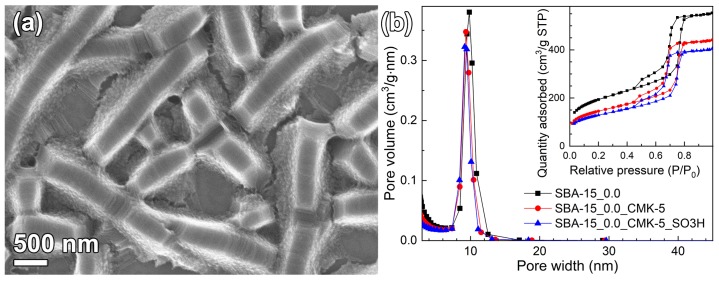
SEM micrograph of (**a**) SBA-15_0.0_CMK-5, and (**b**) pore size distributions and physisorption isotherms of SBA-15_0.0, SBA-15_0.0_CMK-5, and SBA-15_0.0_CMK-5_SO3H.

To study the versatility of the DiG films, an SBA-15_0.0 film was functionalized with a thin carbon layer on the pore walls, similar to CMK-5 [24]. To form the carbon layer, the films were exposed to furfuryl alcohol vapor, instead of the commonly used induced incipient wetness impregnation, since the incipient wetness technique yielded carbon aggregates on the film surface (data not shown). As can be seen in Figure 8a, the film morphology was kept during the carbonization process. However, the unit cell was shrinking during the carbonization, most probably due to densification at 800 °C. Nitrogen adsorption (Figure 8b) indicated that the carbon infiltration resulted in a ~2 Å thick coating, seen as a reduced surface area and pore size compared to the parent SBA-15_0.0, see Table 1. The silica/carbon film was further functionalized by exposure to H_2_SO_4_, which resulted in a 0.191 mmol/g of sulfonic acid sites.

### 3.5. Catalytic Performance

**Figure 9:**
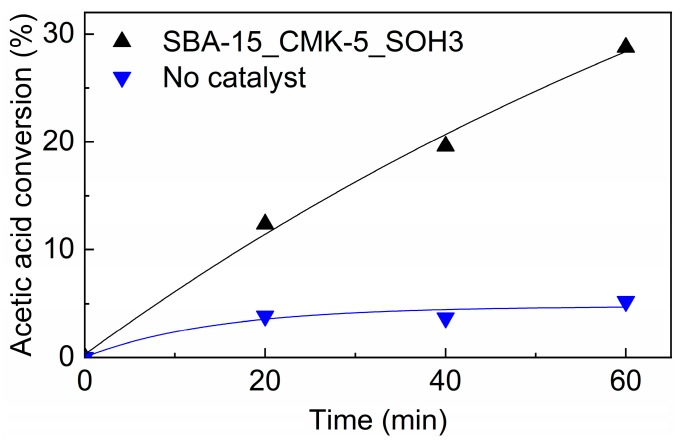
Conversion of acetic acid in the esterification reaction with ethanol at 80 °C, with and without a DiG film catalyst.

To confirm the success of carbon infiltration and functionalization, a 100 mm silicon wafer coated with an SBA-15_CMK-5_SO3H DiG film was prepared. This model system was then tested as a catalyst for esterification of acetic acid and ethanol. The conversion of acetic acid with and without a catalyst is presented in Figure 9. When the film was used as a catalyst, nearly 30 % of the acetic acid was converted within one hour, which was a significant increase compared to the ~5 % conversion when no catalyst was used.

**Figure 10:**
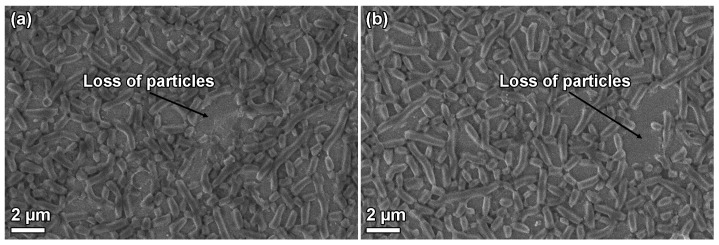
SEM micrographs of SBA-15_0.0_CMK-5_SO3H after (**a**) one cycle and (**b**) two cycles in the esterification reaction.

The catalytic reaction was repeated with a new solution after cleaning the catalyst with water and ethanol. An acetic acid conversion of 11.5 % was obtained in the second cycle. The morphologies of the used films are shown in Figure 10. A majority of the film was intact, except for some areas where particles are removed.

## 4. Discussion

### 4.1. Film Formation

This work shows that it is possible to synthesize DiG films with various thicknesses, which is of interest from an application view point, especially since the films are formed as separate particles with easily accessible pores, also close to the substrate. It has previously been shown that NH_4_F can be used to control the morphology of SBA-15 particles [10,12], and it is apparent from Figure 2 that the film thickness is correlated to the particle width when the salt concentration is altered. This shows that the films are affected by the solution conditions in the same ways as the particles, indicating a similar formation mechanism.

The data shows that film growth occurs when the substrates are added to the synthesis solution during the formation of the siliceous network (Figure 3). At this stage, the micelles are still spherical and the hexagonal ordering has not yet started [28]. This indicates that the films are formed by the condensation of silica species directly on the substrate, and not formed by the deposition of pre-synthesized particles. This is further corroborated by the cross-section images of the films in Figure 1.

It is clear from Figure 5 that proper substrate functionalization is needed to achieve the desired DiG film growth. The densest films are synthesized on hydrophobic substrates, functionalized with octadecyl or methyl groups, while the more hydrophilic substrates show no or poor films consisting of sparsely spaced particles in a tissue phase (Figure 5c–e). Mesoscopic simulations have shown that P123 can form hemispherical structures on hydrophobic surfaces [30]. These structures, where the hydrophobic core is in contact with the substrate, and the PEO brushes are directed towards the aqueous solution, can act as nucleation sites for growth of the film, resulting in a dense packing of particles (Figure 5f,g). Liu et al. showed that non-ionic triblock copolymers (P105) can coat hydrophobic surfaces, while micellar structures can adsorb on a hydrophilic surface [31], which is in good agreement with the simulations. The particle growth on the hydrophilic substrates with silanol groups or P123 (Figure 5a,b) can be the result of adsorbed micelles bound to the substrate through silica polymerization.

The stability of the films is shown in the surfactant removal section and also through the catalytic reaction. It is apparent that the films can sustain calcination at 550 °C, ethanol extraction for 24 h at 78 °C, or ultrasound sonication in methanol at least 5 min, confirming that the particles adhere well to the substrates. During the catalytic reaction, the films were submerged in a stirred, aqueous solution for 1 h per cycle. During the first cycle, some particles detaches from the substrate, although the vast majority of the film remains intact. The loss of particles suggests variation in adhesion among the particles, perhaps due to contaminants present at the particle/substrate interface causing incomplete attachment of the half hexagon prism to the substrate. The possibility to use various surfactant removal techniques enables functionalization through co-condensation, as the functional groups can be removed upon calcination. It also allows post-functionalization since the ethanol extraction yields more silanol groups after micelle removal [32]. H_2_O_2_ oxidation has been shown to be an efficient method for removing P123 and other organic groups from SBA-15 [11,33]. It also removes the DiG film from the substrate. The reason for this can be decomposition of the functional organic layer between the film particles and the substrate.

### 4.2. Functionalization

During the direct sulfonation of SBA-15, it is apparent that the addition time of the MPTMS + H_2_O_2_ strongly affects both the morphology and acidity of the final material, as seen in Figure 7 and Table 1. For SBA-15_0.0, the optimum addition time is 4 h, which yields a material with relatively high acidity and the desired morphology of both films and particles. Adding the reagents as early as 1 h into the synthesis strongly affects the particle formation, and small aggregated particles are formed, while an addition time of 2 h yields a narrower platelet morphology compared to the non-functionalized material. This is most likely due to the fact that the particles at these time points are not completely formed [28]. During the co-condensation of MPTMS, MPTMS will condense on the silanol groups in the siliceous network [34] and hinder further silica condensation at these sites. One hour into the reaction, the siliceous network is forming, but the hexagonal ordering of the micelles has not started. At this time point, particle formation is governed by the addition of MPTMS silica species. When the MPTMS + H_2_O_2_ is added after 2 h, the hexagonal framework has formed, but the particles are still growing through side-by-side attachment of the silicated micelles. The MPTMS attaches to the surface silanols of the silica, and therefore locks the surface from further condensation, resulting in narrower platelets. When the MPTMS + H_2_O_2_ is added after 4 h or directly prior to the hydrothermal treatment, the particle morphology is already set, and no alteration is visible in the SEM micrographs (Figure 7e,g). However, the acidity of these samples is different, which is likely related to the silica condensation also progressing after 4 h [28]. Such condensation enables larger amounts of hydrolysed MPTMS to bind to the silica particles, compared to when the condensation of particles is completed. This is in good agreement to the results by Nassor et al. [35], who showed that it is easy to wash away the MPTMS when it is added at a late stage of the synthesis.

The difference in physicochemical properties of SBA_0.0_no DS and SBA-15_0.0_carbon templates is due to the different methods for removal of the micelles, which were ethanol extraction and calcination, respectively. No external carbon features were detected when the furfuryl alcohol vapor method was used to coat the SBA-15 (Figure 8a). This method yielded a 2 Å thick carbon layer in the mesopores after carbonization (Table 1), without generating additional plugs in the pores (Figure 8b). Hence, the method of using furfuryl alcohol vapor to form CMK-5 structures in mesoporous SBA-15 films shows promising results, which also broadens the application range of DiG films.

### 4.3. Catalytic Performance

Introducing sulfonic acid groups in the mesoporous films result in a substantial increase in the catalytic performance of the film. A conversion of ~5 % of acetic acid during 1 hour of reaction, when no sulfonic acid groups are present, is in good agreement with other studies [13]. This value is boosted by a factor of approximately 6 times when sulfonic acid groups are present in the pores. The results confirm that the mesoporous silica DiG films are successfully coated with carbon using the evaporation technique and that the pores are accessible for both functionalization and catalytic reactions.

SEM micrographs of the used catalyst show that the film is intact after 1 h of reaction, except for a minor particle loss. It is well known that silica dissolves in water, and this reaction may result in loss of particles that have formed with defects towards the substrate. There is no observable additional particle loss after the second reaction cycle, indicating that the remaining particles are well-adhered to the substrate. The catalytic activity decreases after the first reaction cycle. Other studies of esterification reactions using sulfonated carbon catalysts show a similar trend [36,37], and the reduction can be attributed to loss of active sites of the catalyst, either by desorption of sulfonic groups [37], or formation of sulfonic esters on the catalyst surface [38].

## 5. Conclusions

We have shown that monodispersed SBA-15 particles with various aspect ratios can be grown onto silicon wafers using the DiG method. The film thickness follows the particle width and can be tuned between 80 nm and 750 nm by changing the NH_4_F concentration in the synthesis solution. The addition of salt affects the material formation rate, and therefore the substrate addition time must be adjusted so that the substrates are added during the formation of the siliceous network. It has been concluded that hydrophobic substrates are required for a dense film growth, but that substrates with surface silanols or P123 can bond smaller particles to the surface. The film growth is consistent over surfaces larger than 75 cm^2^, and it is possible to coat rough substrates.

We have also shown that the films can be functionalized by co-condensation of MPTMS+H_2_O_2_, but the addition time of the functional reagents must be adjusted so as to not affect the film morphology. The reagents must be added after the formation of the hexagonal order, during the final condensation, to yield a material with high acidity and accessible pores. A CMK-5 structure can also be formed in the films after exposure to furfuryl alcohol vapor. The sulfonated version of the CMK-5 film was shown to work as an efficient catalyst in the esterification reaction of acetic acid and ethanol, showing the accessibility to the pore system, even though the pores are perpendicular to the substrate, due to the separation of the grown particles. The film is stable upon the catalytic reaction, with only a minor loss of particles. Recycling experiments show, however, reduced catalytic activity after the first cycle. As an outlook, one can imagine DiG film growth on other substrates than Si-wafers, e.g. glass or titanium, and utilizing these films as catalyst hosts or drug carrying coatings for implants.

## Figures and Tables

**Table 1:** Physiochemical properties and acidity of the functionalized materials.

Material	Specific Surface Area (m^2^/g)	Pore Size (nm)	Pore Volume (cm^3^/g)	Unit Cell Parameter (nm)	Acidic Sites (mmol/g)
SBA-15_0.0_no DS ^a^	920	11.2	1.19	13.6	0.003
SBA-15_0.0_1DS	948	8.9	1.10	11.2	0.024
SBA-15_0.0_2DS	898	10.2	1.11	12.6	0.009
SBA-15_0.0_4DS	870	10.4	1.07	13.6	0.020
SBA-15_0.0_20DS	951	11.3	1.22	14.1	0.008
SBA-15_0.0_carbon temp ^b^	697	9.8	0.86	12.9	-
SBA-15_0.0_CMK-5	521	9.4	0.68	12.4	-
SBA-15_0.0_CMK-5_SO3H	462	9.3	0.63	12.5	0.191

^a^ Reference SBA-15_0.0 from the same batch as the directly sulfonated materials. ^b^ SBA-15_0.0 template for the carbon infiltration.

## Supplementary Materials

The following are available online at https://www.mdpi.com/2079-4991/9/4/562/s1, Methods for substrate functionalization, methods for surfactant removal, Figure S1: Pore size distributions, physisorption isotherms, and small angle x-ray diffractograms for materials synthesized with NH4F/P123 molar ratios of 0.0–1.8, Figure S2: Pore size distributions, physisorption isotherms, and small angle x-ray diffractograms for direct sulfonated SBA_0.0s, Table S1: Physiochemical properties and acidity of materials synthesized with NH4F/P123 molar ratios of 0.0–1.83.
