# Supplementary material for: Growth and Functionalization of Particle-Based Mesoporous Silica Films and Their Usage in Catalysis
Source: Nanomaterials (Basel). 2019 Apr 6;9(4):562. doi: 10.3390/nano9040562 (PMC6523614; doi:10.3390/nano9040562)
Supplement: Supplementary file 1 [file nanomaterials-09-00562-s001.pdf]

Growth and functionalization of particle based mesoporous silica films and their usage in catalysis

Pei-Hsuan Wu, Peter Mäkie, Magnus Odén and Emma M. Björk*

[1] Nanostructured Materials, Department of Physics, Chemistry and Biology (IFM), Linköping University, 581 83 Linköping, Sweden
[2] Institute of Inorganic Chemistry II, University of Ulm, Albert-Einstein-Allee 11, 890 81 Ulm, Germany
* Correspondence: emma.bjork@liu.se

Supplementary Material

Substrate functionalization

Methyl substrates

Cleaned substrates were placed in a sealed vessel with few droplets of TMCS at room temperature for 15 min. The vessel was then kept open for the residue of TMCS evaporated for 20 min prior to use.

Amino substrates

The APTMS functionalization followed a modified route from Du [1]. Cleaned substrates were immersed in 1% APTMS in ethanol at ambient temperature for 2 h followed by through rinsing with 95% ethanol. The substrates were stored in 95 % ethanol and were washed with deionized water and dried under a continuous stream of N_2 gas prior to film deposition.

Thiol substrates

Substrates were functionalized with MPTMS according to the protocol by Shiku et al. [2]. Cleaned substrates were soaked in a solution with 10 mM MPTMS in benzene for 8 h at ambient temperature. The substrates were then washed with benzene, ethanol, and deionized water under sonication.

Sulfonic acid substrates

Thiol functionalized substrates were prepared as described above. The substrates were then immersed in a 1.0 M H_2O_2 solution for 30 min to oxidize the thiol terminal group to sulfonic acid and then washed with deionized water.

P123 substrates

P123 substrates were prepared following the protocol by Wooten et al. [3]. A drop of glycerol was first added in an acetone solution with 0.696 mM P123 under constant stirring, and an equimolar amount of 1,6-diisocynanatohexane was then added in the mixture. As-prepared clean substrates were dip-coated in the mixture and aged at 120 °C overnight.

Template removal

Ethanol extraction

Ethanol extraction was performed using a modified procedure of Li et al. [4]. 0.2 g of as synthesized SBA-15 powder and films was immersed in 150 ml of 99.5 % ethanol under reflux at 78 °C for 24 h. The material was then filtered and dried at room temperature.

H_2O_2 oxidation

H_2O_2 oxidation of the P123 was performed following the route protocol by Johansson et al. [5]. One synthesis batch, including films, was mixed with 120 g H_2O_2 solution under reflux at 100 °C for 24 h. The material was then filtered and dried at room temperature.

Methanol sonication

Methanol sonication was performed according to the route described by Pirez et al. [6]. 100 mg of SBA-15 and film samples were covered with methanol in a sealed in polypropylene vessel and immersed in an ultrasound bath at ambient temperature for 5 min. After the post-extraction, the material was rinsed with methanol, collected by filtration, and finally dried in an oven at 80 °C.

Nitrogen sorption and x-ray diffraction data for SBA-15 particles

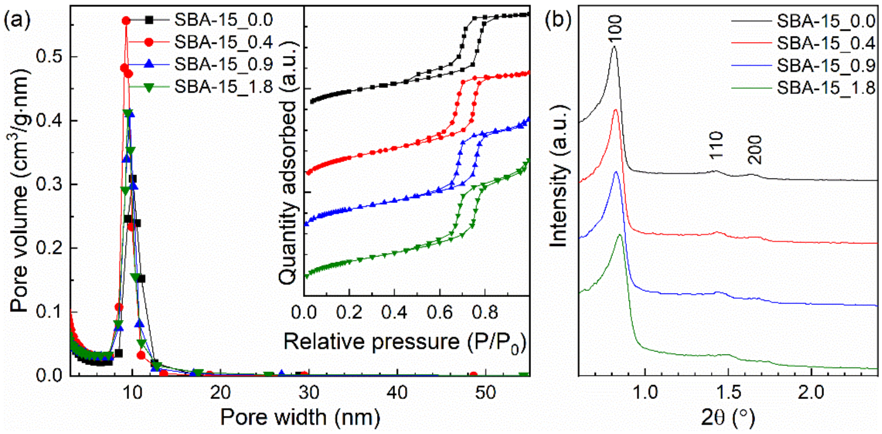

Figure S1. (a) Pore size distribution and physisorption isotherms, and (b) small angle x-ray diffractograms of materials synthesized with NH_4F/P123 molar ratios of 0.0 – 1.8.

Table S1. Physiochemical properties and acidity of materials synthesized with NH_4F/P123 molar ratios of 0.0 – 1.83.

Sample	Specific surface area[a] (m^2/g)	Pore size[b] (nm)	Pore volume[c] (cm^3/g)	Unit cell parameter (nm)
SBA-15_0.0	675	10.1	0.87	12.4
SBA-15_0.4	784	9.3	0.97	12.3
SBA-15_0.9	610	9.7	0.93	12.2
SBA-15_1.8	606	9.5	1.02	11.9

[a] Calculated with the BET method at P/P_0 = 0.10 – 0.20.

[b] Calculated using the KJS method on the adsorption isotherm.

[c] Estimated at P/P_0 = 0.98.

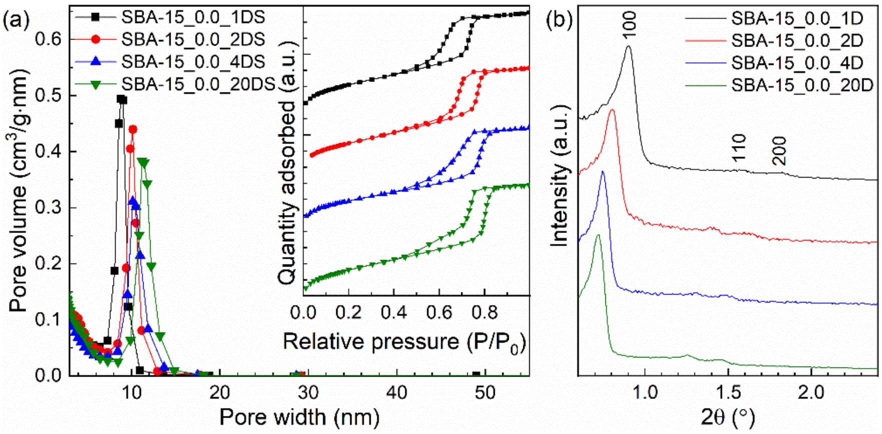

Figure S2. (a) Pore size distribution and physisorption isotherms, and (b) small angle x-ray diffractograms of direct sulfonated SBA_0.0s.

References

1. Du, H. Microcantilever surface modification for chem-/bio-sensing. PhD thesis, College of engineering and science, Louisiana tech university, 2007.
2. Shiku, H.; Uchida, I.; Matsue, T. Microfabrication of Alkylsilanized Glass Substrate by Electrogenerated Hydroxyl Radical Using Scanning Electrochemical Microscopy. *Langmuir* **1997**, *13*, 7239-44.
3. Wooten, C.; Kaitlyn, M.; Koganti, V.R.; Zhou, S.; Rankin, S.E.; Knutson, B.L. Synthesis and Nanofiltration Membrane Performance of Oriented Mesoporous Silica Thin Films on Macroporous Supports. *ACS Appl. Mater. Interfaces* **2016**, *8*, 21806-15.
4. Li, Y.; Sun, N.; Li, L.; Zhao, N.; Xiao, F.; Wei, W.; Sun, Y.; Huang, W. Grafting of Amines on Ethanol-Extracted SBA-15 for CO_2 Adsorption. *Materials* **2013**, *6*, 981-999.
5. Johansson, E.M.; Cordoba, J.M.; Oden, M. The Effects on Pore Size and Particle Morphology of Heptane Additions to the Synthesis of Mesoporous Silica SBA-15. *Microporous Mesoporous Mater.* **2010**, *133*, 66-74.
6. Pirez, C.; Wilson, K.; Lee, A.F. An Energy-Efficient Route to the Rapid Synthesis of Organically-Modified SBA-15 Via Ultrasonic Template Removal. *Green Chem.* **2014**, *16*, 197-202.
